# Supplementary material for: Are carbon emissions trading and green financial instruments synergistic? -Comprehensive quantitative research based on content analysis
Source: PLoS One. 2024 Mar 7;19(3):e0298601. doi: 10.1371/journal.pone.0298601 (PMC10919601; doi:10.1371/journal.pone.0298601)
Supplement: S1 Data — (ZIP) [file pone.0298601.s001.zip › Data/地级市Z_F工作报告.zip!/─■╧─/╬Γ╓╥╩╨/└┤╫╘╣1⁄2╓┌║┼í╛┬φ┐╦╩2╛▌═°í┐_2009.docx]

各位代表：

现在，我代表市人民政府向大会作政府工作报告，请予审议，并请市政协委员和其他列席人员提出意见。

一、2008年工作回顾

2008年是我市跨越式发展攻坚克难、大步迈进的重要一年。一年来，在自治区党委、政府和市委的坚强领导下，在市人大、政协的监督支持下，市政府团结带领全市各族人民深入贯彻党的十七大和十七届三中全会精神，认真落实科学发展观，开拓创新，务实苦干，较好地完成了市三届人大一次会议确定的目标任务。预计完成地区生产总值168.9亿元，增长13.1%；完成地方财政收入13.3亿元，可比增长41%；完成全社会固定资产投资130亿元，增长59%；城镇居民人均可支配收入和农民人均纯收入分别达到11400元和4100元，增长17.8%和13.5%。

工业经济稳步攀升。深入实施“工业强市”战略，坚持以“两城五园”为抓手，加快结构调整和技术创新，狠抓项目建设、产业升级等关键环节，全面落实国家和自治区税费减免、电价优惠等政策，千方百计稳固符合产业政策与环保要求的生产能力，工业经济在外部环境发生重大变化的情况下，继续保持平稳增长。预计完成工业总产值275亿元，增长18%，规模以上增加值69亿元，增长15%。年初确定的52个重点项目，开工建设48个，完成投资61亿元。庆华100万吨煤焦化、太阳山风电一期、大唐大坝2×60万千瓦机组、娃哈哈2万吨全脂奶粉等24个项目建成投产；阳光矿业永安120万吨矿井、青铝2×30万千瓦自备电厂、萌生水泥日产2000吨新型干法水泥生产线、同心麦克威尔300吨无毛绒、科冕公司万吨葡萄酒、吴忠卷烟厂技改扩建等项目顺利推进。地方工业完成增加值42亿元，增速连续三年保持在20%以上，占全市工业的比重首次超过50%。“两城五园”基础设施进一步完善，产业聚集和项目承载能力明显增强。各园区新入园企业35家，园区完成工业总产值227亿元，占全市工业总产值的82.5%。节能减排工作强力推进，循环经济发展取得新的进展。万元GDP能耗下降6.46%，主要污染物二氧化硫削减2599吨，削减率为2.4%，化学需氧量削减6284吨，削减率为12%。

农村经济扎实推进。坚持以增加农民收入为核心，全面落实各项强农惠农政策，调整优化农业结构，推进“十大产业基地”建设，优势特色产业规模不断扩大。预计全年完成农业总产值53.3亿元，增长7.5%。兑现各种惠农资金2.9亿元；优质粮食面积70万亩，粮食总产量达8.6亿公斤；针对“三鹿奶粉”事件冲击，及时协调杭州娃哈哈集团订购库存合格奶粉，动员各级干部职工带头认购放心奶，并加强政策扶持，使奶产业平稳渡过危机，保持稳步发展。新增奶牛2.9万头，存栏19.1万头；肉牛出栏25万头；羊饲养量451万只。新增设施农业10.3万亩，超过历年面积之和，总面积突破20万亩；高酸苹果、红枣、酿酒葡萄、甘草分别新增12万亩、4万亩、7.2万亩和21.3万亩。培育市级以上农业产业化重点龙头企业96家，农村专业经纪合作组织发展到286家，有效拉动农业增效、农民增收。当年建成农村饮水安全工程6处，解决了8万农民饮水安全问题。新建改建农村公路445公里，超过计划任务145公里。山区县（区）首次和川区同步大搞农田水利基本建设，全市共砌护渠道731公里，新建改造水利设施2800多座，新修旱作“三田”5.3万亩，改造中低产田9万亩，青铜峡市夺得“黄河杯”。建成同心韦州南滩、盐池南苑新村等生态移民村5个，开发节水灌溉6万亩，搬迁安置移民4.7万人。输出劳务24万人次，实现劳务收入11.8亿元。完成各类造林68.7万亩。新建塞上农民新居示范点9个，整治改造旧庄点76个，建成“一池三改”户用沼气池8778座，改造危窑危房1820户。农村面源污染得到有效治理。

城乡建设大步迈进。抢抓自治区成立五十周年和地级吴忠市成立十周年的有利时机，加快推进城镇基础设施建设，强力推进市区与小坝西移东扩。加强城乡规划管理，提升了人居环境和投资环境。全市共实施城市建设项目368个，完成投资66亿元，城市化率达到42.6%，比2007年提高2个百分点。市区城市防洪排涝工程、新汽车站、老城区供热管网改造及唐风、秦韵、开源三个广场等建成使用。教育园区、民俗博物馆、伊斯兰文化中心及黄河公路大桥等项目开工建设，黄河中上游一座独具特色的滨河生态水韵城市已初步展现在人们面前。青铜峡市实施罗家河综合整治、火车站改造、沿河快速通道等工程，东扩步伐明显加快，城市品位大幅提升。盐池县实施县城五大出入口改造、供排水管网改造、城市绿化等项目，新增城市绿地面积7600多亩，县城面貌焕然一新。同心县实施清水河综合治理、省道101线改道等工程，大步推进新区建设，新建成的5000亩水面，使中部干旱带凸现高塬平湖新景观。红寺堡开发区实施建材市场和盐兴路城区段改造项目以及环城林网建设，城市聚集功能进一步增强。滚红高速公路正式开工，省道101线吴忠至金积、明珠路至早元路、国道307线盐池县城至王圈梁、盐中高速公路孙家滩连接线、惠平公路太阳山段改建等一批国省干道工程相继竣工通车。深入推进“两大工程”，建立城乡环境整治长效管理机制，不仅使城乡建设管理中一些多年来没有解决的难题得到根治，城乡面貌呈现出前所未有的崭新形象，而且使干部得到了锻练，群众得到了实惠。全面启动“国家园林城市”和“全国卫生城市”创建活动，市区和盐池县双双通过自治区园林城市和卫生城市创建验收。

第三产业凸现活力。大力整合商贸资源，加快区域性市场基础配套建设，市区商贸核心区改造、再生资源市场建设全面完成，涝河桥牛羊肉批发市场、同心羊绒市场等专业市场功能明显提升。深入实施“万村千乡市场工程”，新建改建农家店260个，配送中心6家，农村市场网络不断完善。物流配送、交通运输、电子商务等商贸流通业及金融、住宿、餐饮等服务业层次进一步提升，日常人均消费持续增长。预计全年实现社会消费品零售总额37亿元，增长19%，增幅创近年新高。启动“中国优秀旅游城市”创建活动，编制了《黄河青铜峡大峡谷旅游区规划》，完善黄河十里长峡等旅游基础设施，完成盐池苏维埃政府纪念馆等旅游景点建设，加快发展以农家乐旅游为主的城市郊区旅游和民俗风情旅游、革命老区红色旅游等特色旅游项目，全年接待游客136万人次，实现旅游收入4.6亿元，增长28%。制定出台了《关于促进吴忠市房地产业快速健康发展11条措施》，全市共完成商品房销售面积61万平方米，实现销售额11.4亿元，分别增长16.9%和39.9%，房地产业呈现新的活力。

改革开放不断深化。深入推进企业产权制度改革，完成益木公司、瑞特纸业、吴忠水泥、商业车队等企业职工身份置换，解决了10家企业历史遗留问题。积极稳妥地推进事业单位分类改革。利通区教师实行全员竞聘上岗；完善专业技术人员职称评审机制，为106名农村实用人才评定了职称，农村实用人才职称评定工作走在全区前列。深化行政管理改革，实行人员编制动态管理，推行政府投资项目代建制管理模式。完善国库集中支付、政府采购和部门预算制度改革，落实乡财县管制度，化解乡镇债务2.2亿元。农村新型金融机构建设迈出了实质性步伐，设立了滨河村镇银行和农村互助资金组织。启动了建立保障和促进科学发展土地管理新机制试点工作，进一步规范土地市场，合理配置土地资源。认真实施土地开发整理项目，确保耕地总量动态平衡，全市耕地保有量达到469万亩。组团参加了西洽会等投资贸易洽谈会，成功举办了第二届中国(吴忠)中小学生书法节、自治区成立50周年吴忠成就展、地级吴忠市成立10周年成就展、中央电视台“激情广场大家唱”吴忠专场演出、陕西秦腔名家演唱会及特色农副产品展销会等大型活动，积极配合完成重大革命历史题材电影《同心》的拍摄和公映，提高了吴忠知名度和影响力。创新招商机制，优化招商环境，拓宽招商领域，广泛开展招商引资活动，成功引进了浙江盾安集团、华盈集团等59家企业落户吴忠，实际到位资金90.2亿元。

社会事业全面进步。始终坚持教育优先发展战略，加大教育投入，落实项目资金4.2亿元，化解了义务教育债务，新建民族技术学院、吴忠回民中学、同心豫海回民中学等一批学校，改造危房3万平方米，办学条件明显改善。整合教育资源，提升教育教学质量，促进教育均衡发展，“两基”工作顺利通过国家验收。深入推进科技特派员创业行动，科技特派员达到595人，结成利益共同体241个，设施农业、奶牛养殖、优质粮食等新品种、新技术全面推广应用，科技对经济增长的贡献率达到48%。“科技强警”通过国家公安部、科技部的验收，成为西北地区唯一非省会“科技强警”示范城市。加强文化基础设施建设，建成市图书馆。新农村信息化建设顺利通过国家8部委验收，同心县被评为全区新农村信息化建设先进集体，盐池县被命名为文化信息资源共享工程示范县。实施市中医院、同心县中医院、青铜峡市人民医院、盐池县中医院和7个乡镇卫生院改扩建项目，医疗卫生条件不断改善，服务网络不断完善，妇幼保健覆盖面进一步扩大。全面启动和推行城镇居民基本医疗保险、农民新型合作医疗，大力开展农民健康教育与健康促进行动，全面推行“药品三统一”。继续开展计划生育“三无”、“一无”乡镇创建活动，扎实推进“少生快富”工程和奖励扶助制度，人口自然增长率为9.45‰，计划生育率为94.49%。组织防空袭及消防、防震减灾演练活动，不断加强消防安全、防震减灾、国防动员教育和人民防空工作，建成吴忠市民兵预备役综合训练基地，军民共建和“双拥”工作顺利推进，吴忠市首次争创全国“双拥模范城”获得成功。全面贯彻落实党的民族宗教政策，民族团结、宗教稳定。气象、统计、广播电视、妇女儿童、物价、残疾人事业、住房公积金等工作有了新的发展。

民生改善全面推进。制定出台了《关于大力推进全民创业的实施意见》，建立小额担保贷款联动机制，共发放小额担保贷款1774万元，创办小企业329个，创造就业岗位3000多个。落实积极的就业政策，城镇新增就业1.2万人，城镇登记失业率控制在4.2%以内。加强社会保障体系建设，建立完善失业、工伤、医疗等保险制度，健全城乡居民最低生活保障、失地农民社会保障及村干部养老保险制度，推行农村社会养老保险试点，城乡社会救助体系不断完善，城乡低保人数达到9.3万人，连续两次提高救助标准。新建经济适用住房9.6万平方米，廉租住房456套，将符合条件的554户城市低收入家庭纳入廉租住房租赁补贴，累计发放租赁补贴171万元，有效缓解了城市低收入家庭住房困难。募集社会资金330多万元，帮助1069名特困家庭大学新生上学。扎实推进扶贫帮困、救灾救助工作，发放救灾资金600万元，救灾粮10.5万公斤，救助灾民11万人。社会各界共向四川地震灾区捐赠款物2550万元，援建安置房400套。

民主法制不断加强。坚持用社会主义核心价值体系引领社会风尚，大力倡导和践行社会主义荣辱观。深入开展思想道德建设年活动，加强社会公德、职业道德、家庭美德、个人品德和未成年人思想道德建设，涌现了“优秀检察官”马俊、“缉毒英雄”苗卫明等一批先进典型，弘扬了社会正气。深入开展“五五”普法教育，全民法制意识和政府依法行政、依法办事水平明显提高，中期督导验收获全区优秀等次。各级政府自觉接受人民代表大会及其常务委员会的依法监督和人民政协的民主监督，加强同群众团体组织的联系，认真办理人大代表议案、建议和政协委员提案。加强机关效能建设，以建章立制、强化问责为抓手，加大督查督办和效能问责，行政效能明显提高，政务环境明显优化。以维护司法公正、提高案件执行率为抓手，大力优化司法环境。以全面清理党政机关及公务人员托欠银行贷款为突破，切实优化金融环境。全面推行政务、村务、校务、厂务公开，实行便民“一站式”服务；扩大市政务大厅进厅部门和办理事项，工商、税务、质监等区直部门集中进驻，新增审批服务事项16项，办件量增加1倍。红寺堡“阳光”村务经验在全区推广。高度重视信访工作，坚持和完善市级领导信访接待日制度，在全区率先建立了乡镇矛盾纠纷排查调处中心、便民服务中心；各县区（市）认真开展县委书记大接访活动，切实解决了一批人民群众反映的实际问题。全面开展平安创建活动，狠抓社会治安综合治理，严厉打击各类违法犯罪活动，实现了“命案必破”，破案率提高5.7个百分点。认真开展人民禁毒战争，毒品问题重点整治工作得到巩固和加强。加强廉政建设，强化审计监督和行政效能监察，着力从源头上预防和惩治腐败，严肃查处了一批违纪行为。加强食品安全、药品安全、生产安全工作，全面落实安全生产责任制，加强日常监管和专项整治，各项安全生产指标控制在自治区下达控制目标以内，社会秩序祥和安定。

各位代表！刚刚过去的一年，是我们积极把握国务院《关于进一步促进宁夏经济社会发展的若干意见》（以下简称国务院《若干意见》）出台和自治区成立五十周年、地级吴忠市成立十周年的有利形势，借势而上，全力突破，在抢抓机遇中大干快干的一年；是面对国内自然灾害及我市五十年不遇旱情和国际金融危机冲击，沉着思考，积极应对，在攻坚克难中大步前进的一年；是紧紧围绕自治区第十次党代会和市委三届四次全体会议确定的跨越式发展目标，谋发展、促跨越、抓落实，在加快发展中变化最大、最深刻的一年。一年来，在市委的正确决策和坚强领导下，我们坚持以解放思想为先导，深入开展“思想再解放、市情再认识、发展大跨越”大讨论活动，通过“两大工程”、农田水利基本建设等实践活动的历练，在城乡面貌发生深刻变化的同时，干部群众干事创业的激情更加高涨；我们坚持以人为本、和谐惠民的发展理念，千方百计解决关系民生改善和社会稳定的问题，从点点滴滴着手为百姓谋福祉，在人民群众得到更多发展实惠的同时，党群、干群关系更为密切融洽；我们坚持加强对县区（市）工作的领导和指导，注重调动县区（市）的发展积极性，在形成不甘落后、争创一流竞争局面的同时，县域经济发展活力更趋强劲；我们坚持狠抓效能建设不放松，大力简政放权，在政务、司法、金融三个环境建设取得实质性进展的同时，服务跨越式发展的能力得到进一步增强；我们坚持自觉接受人大、政协及社会各界的监督，主动维护民族团结，保持宗教和顺，充分调动一切积极因素，保稳定、促和谐，在形成加快发展合力的同时，科学决策、民主决策的水平有了进一步提高；我们坚持紧紧依靠和团结全市各族人民、各级干部抓项目、争资金，抓建设、促发展，在项目建设取得重大进展的同时，凝聚力、执行力得到进一步强化。回顾一年来取得的成绩，我们深深感到：这是自治区党委、政府亲切关怀和市委科学决策、正确领导的结果，是市人大、市政协依法监督、大力支持的结果，是全市130万各族人民团结一致、共同努力的结果。在此，我代表市人民政府，向为吴忠发展做出无私奉献、付出辛勤劳动的全市人民致以亲切问候和崇高敬意！向给予政府工作大力支持的人大代表、政协委员、各民主党派、工商联、无党派人士、各人民团体、离退休老同志和社会各界人士，向为吴忠改革发展稳定做出积极贡献的公安干警、驻吴人民解放军、武警官兵，向所有关心、支持吴忠发展的中央及区属驻吴单位，表示诚挚祝愿和衷心感谢！

认真总结过去的工作，我们也清醒地看到，全市经济社会发展中还存在不少困难和问题。主要表现在：一是市域经济快速增长的基础还不牢固，山川之间、城乡之间发展不平衡，山区扶贫开发任务依然十分繁重；二是工业结构调整步伐还不快，新上大项目少，以企业为主体的技术创新体系还没有形成，技改、节能投入不足，工业产品单一、耗能较高的局面还没有根本改善；三是农业特色产业规模扩张不快，龙头企业带动力不强，资金、技术、销售等瓶颈制约问题仍未有效解决，农民增收渠道还不宽；四是财政基础薄弱，新增财源和骨干财源少，刚性支出增多，用于解决民生等问题的财力少；五是城市建设融资难，解决历史遗留问题困难较多；六是人民群众关心的一些热点、难点问题还没有解决好，人民群众还有很多合理期待一时难以实现；七是政府效能建设、政务环境、司法环境还需进一步加强和改善。对于这些问题，我们将采取更加有力的举措，认真加以解决。

二、2009年工作任务

今年是新中国成立60周年，也是积极应对金融危机冲击，加快跨越式发展的关键之年。如何在比较困难的情况下，充分利用各种有利条件，把严峻考验转化为发展机遇，是我们面临的巨大挑战。我们必须以更加坚定的决心、更加昂扬的斗志、更加有力的措施，扎实工作，渡过难关，把吴忠的发展不断推向前进。今年政府工作的总体思路是：全面贯彻党的十七大和十七届三中全会、自治区十届七次全体（扩大）会议和市委三届六次全体（扩大）会议精神，深入学习实践科学发展观，认真落实国务院《若干意见》，解放思想，抢抓机遇，深化改革，扩大开放，着力推进经济结构调整和发展方式转变，着力推进工业“两城五园”、农业“十大产业基地”建设和现代服务业发展，着力改善民生，加快推进城市化进程，加快建设社会主义新农村，全面加强文化建设、社会建设和民主法制建设，促进社会和谐，奋力推动全市经济社会又好又快发展，以优异成绩向新中国成立60周年献礼。

全市经济社会发展的主要预期目标和约束性指标是：地区生产总值增长12%；地方财政一般预算收入增长12%；全社会固定资产投资增长25%；社会消费品零售总额增长13%；城镇居民人均可支配收入和农民人均纯收入分别增长10%和6%；人口自然增长率控制在10‰以内；城镇登记失业率控制在4.3%以内；万元GDP能耗下降4%，二氧化硫和化学需氧量分别削减11%和7%。

确立这样的预期目标，是充分估计和把握今年经济形势的复杂程度和国家“保增长”的总体要求，深入分析国家宏观政策走向、全区经济发展的走势及周边地市的发展态势，结合我市实际提出的。既考虑了加快实现跨越式发展目标的现实需要，又考虑了实现目标的可能性、复杂性与艰巨性，总体上是可行的。挑战前所未有。当前，国内外经济环境还在不断变化，国际金融危机尚未见底，对国内经济特别是吴忠发展的影响难以预测，完成今年目标任务的不确定因素很多，城乡居民实现稳定增收、地方财政收入实现预期增长、就业保持稳定都将面临巨大挑战，有可能要遭遇改革开放以来最为严峻的考验。机遇前所未有。在看到危机的同时，我们也要危中见机。我国发展的重要战略机遇期没有改变，经济发展的基本面是好的，发展潜力是巨大的，中央保“8”、自治区争“10”的经济增长目标及其一系列政策措施是积极有力的，预示着今年经济发展大环境总体上会越往后越有希望。同时，国务院《若干意见》和扩大内需政策叠加产生的倍增效应，将在我们的积极争取下逐步显现，为我们加快发展、实现“保增长”目标提供支持。信心非常关键。当前危机是传统发展模式之“危”，是科学发展之“机”，是对我们发展信心的巨大考验。多年来，我市经济持续发展，基础设施不断完善，工业化、农业产业化、城市化进程不断加快，产出能力正在迅速提高，多年积蓄的发展能量将逐步释放，有着强劲的内在发展动力；全市社会稳定，上下齐心，人心思进，干劲十足，蓄积了强大的社会精神动力。只要我们始终坚定信心，积极应对，就能转危为机、化不利为有利，就能将金融危机带来的影响降低到最低程度，就能在逆境中奋力推进我市经济社会又好又快发展。同时，必须牢牢把握好以下几个方面：

一要牢牢把握科学发展这个前提。当前困难不仅是外部环境影响，也是我们经济发展结构性矛盾等深层次问题的集中显现，说到底还是发展不充分的问题。我们必须以学习实践科学发展观活动为动力，全力加快结构调整，把一产调优，二产调强，三产调大，使三次产业内部结构更趋合理、更趋优化。只要我们始终坚持一切从实际出发，把促进当前经济增长与解决长期问题结合起来，切实增强应对危机的预见性、科学性和有效性，牢牢掌握发展特别是经济建设的主动权，就一定能渡难关、上水平，使我市经济社会发展真正转入科学发展轨道。

二要牢牢把握扩大内需这个关键。扩大内需是我们应对当前危机的关键之举。必须以“对事业负责、对人民负责、对历史负责”的紧迫感和责任感，坚持把上争项目、扩大投资、拉动内需作为应对危机的首要任务，紧紧围绕国务院《若干意见》和扩大内需10条措施及相关配套性政策，积极争取项目、资金和政策支持，力争落实和新上一批能带动全局、事关长远的大项目、好项目，通过大投资促进大发展，为可持续发展打牢基础。

三要牢牢把握和谐惠民这个根本。越在困难时期，越不能放松对民生的关注，越不能降低对民生的保障。必须始终坚持把改善民生作为加快发展的出发点和落脚点，牢固树立时时以民生为重，事事以民生为急的政绩观，深入实施“民生计划”，财力再紧再难，都坚持公开承诺为民办实事，着力解决人饮解困、卫生惠民、城乡安居、扶弱助困等民生问题，妥善处理各种利益关系，切实把各项利民、惠民、安民的政策措施落到实处。在推动川区快速发展的同时，更加关注山区的发展，更加重视山区群众的脱贫致富。

四要牢牢把握改革创新这个主线。坚持把改革创新贯穿于应对危机、加快发展全过程，继续深化企业产权制度改革，实现制度、管理、技术创新，使改制企业改彻底、有活力、快发展。积极创造条件，鼓励中小企业、非公有制企业实行产业转型和结构优化升级；制定完善小额担保贷款与创业联动机制，鼓励支持全民创业，让发展的活力充分涌流，创业的激情竞相迸发。

五要牢牢把握财源建设这个基础。积极应对经济增速放缓和增值税政策性转型等对财税工作的影响，高度重视和加强财政工作，积极壮大骨干财源，稳定基础财源，增强公共财政保障能力。牢固树立过紧日子的思想，从支出结构上集中力量办大事，优先支持基本建设和民生改善。严格压缩党政机关及事业单位公务性支出，坚持节俭办事，做到公务支出“零增长”。

在重点工作上，着力抓好以下八个方面：

一、着力提高工业经济发展质量

坚定不移地实施工业强市战略，把保增长和调结构有机结合起来，在调整结构中扩大总量，在技术升级中激活存量，在转变增长方式中提升效益，确保规模以上工业增加值增长13%以上。加强协调服务，力促工业平稳运行。动员全市上下聚焦工业、服务工业、发展工业，调动各种要素促进工业增长。加强对宏观经济形势、市场变化走势分析预测，落实国家、自治区各项扶持政策，及时帮助工业企业协调解决问题和困难，保证企业正常生产。鼓励和引导企业改进管理模式，降低运行成本，提高经营效益。狠抓项目建设，夯实工业发展基础。力促大唐大坝2×60万千瓦火电机组、娃哈哈2万吨全脂奶粉、青鑫碳素2万吨阴极炭块等项目达产达效；完成太阳山风电场二期、昊盛纸业棉浆粕、吴忠卷烟厂技改扩建、盐池萌生水泥技改等项目建设任务，确保年内投产达效；争取盾安金太阳镁业金属镁一期、建材集团水泥、庆华重介洗煤、银星能源多晶硅、金积热电厂、青铝集团自备电厂、红寺堡风电二期等项目全面开工。力争年内新增工业产值45亿元。加大技术改造，推进结构优化升级。鼓励企业引进高新技术和先进实用技术改造提升现有工艺，加速产品研发和更新换代，提高产品档次和附加值。抓好迈科铝业、宁鲁石化、银星能源、庆华集团等工业技改项目，促进骨干企业发展壮大。落实国家促进中小企业发展的政策措施，加强对中小企业的信用担保、技术创新、创业培训等服务；争取国家和自治区相关专项扶持资金和金融信贷支持，加快发展装备制造、粮食加工、食品加工等特色工业；鼓励中小企业、非公有制企业实行产业转型和结构优化升级，向专、新、特、精方向发展，提高市场竞争力和抗风险能力。加快园区建设，筑牢工业发展平台。完善“两城五园”基础配套设施，提升园区产业聚集和项目承载能力。抢抓国家启动宁东三大基地建设的时机，争取政策扶持和项目支持，促进太阳山的资源优势向经济优势转化；完成青铜峡新材料基地控制性详细规划和产业功能区布局，积极申报自治区级工业园区；加快金积工业园区包装印刷城和造纸化工循环经济园建设；继续加快青铜峡嘉宝、盐池东顺、同心羊绒、红寺堡工业园基础配套建设，创新管理机制，优化政策服务，主动承接发达地区产业转移。加大招商引资力度，力争年内完成招商引资80亿元。推进节能减排，着力发展循环经济。认真落实节能降耗和减排目标责任制，支持冶金行业按照国家产业政策，以节能降耗和保护环境为目标，推动铁合金矿热炉向大型化、半密闭、自动化、达标排放的先进炉型发展，促进各项污染物排放达到环保要求。推进庆华循环经济工业园、青铜峡水泥公司低温余热发电及同心、红寺堡污水处理厂、大坝电厂燃煤机组脱硫等项目建设，全力抓好城市燃煤锅炉、乳品行业综合治理，推动节能减排。

二、着力加快农业农村经济发展

紧紧围绕川区率先建成现代农业示范区、山区率先建成节水旱作高效农业示范区的目标，加大农业结构调整力度，加快推进农业“十大产业基地”建设，推进农村改革与发展，促进农业增效、农民增收，力争全年实现农业总产值57亿元，增长7%。一是扩大优质粮食生产。抢抓国家提高粮食最低收购保护价的政策机遇，继续落实各项惠农政策，调整优化种植结构，继续扩大优质粮食种植面积，稳定粮食生产，确保优质粮食种植面积达到80万亩以上，全市粮食总产量稳定在8亿公斤以上。二是积极发展特色优势产业。落实国家、自治区发展畜牧业扶持政策，大力发展奶牛、滩羊和肉牛养殖业，抓好孙家滩、牛首山北麓畜牧养殖带建设，新建扩建各类养殖园区20个；盐池、同心、红寺堡重点发展肉牛和滩羊规模化养殖，不断扩大青铜峡生猪养殖规模，搞好畜禽品种改良和动物疫病防控工作，提高养殖水平。力争年底奶牛存栏25万头，新增6万头；肉牛出栏30万头，较上年增加5万头；羊饲养量达到500万只；生猪饲养量达110万头。农民人均畜牧业收入达到2600元以上。巩固提高利通区金积、青铜峡叶盛、盐池城西滩、红寺堡大河等7个万亩设施农业园区和20个千亩设施农业园区，全市新增设施农业5万亩，高酸苹果3万亩、红枣10万亩、酿酒葡萄5万亩、人工甘草20万亩。三是推进农业产业化经营。实施农业产业化“龙头企业升级工程”，落实相关扶持政策，培育壮大娃哈哈恒枫乳业、塞外香、荣宝园、天予枣业、科冕酒业等企业，促其上规模，上档次，增强带动能力。完善企业与农民的利益联结机制，扩大订单农业面积。通过龙头带动，延伸产业链，提高加工转化率。积极争取国家强农支农项目和资金，提升农业机械化、标准化生产水平。加快无公害农产品、绿色农产品和有机食品示范基地建设及认证工作，成立吴忠市食品检测中心，年内完成无公害产品基地认定2个，产品认证10个，绿色食品认证3个，建成孙家滩工厂化育苗中心和市区绿色农产品物流配送中心，每个县至少组建一个农产品物流公司，全市新发展农民专业化合作组织30个，建立农村信息服务站40个，初步建立满足现代农业发展的农产品销售流通体系，不断推进农业产业化经营。四是加强农村基础设施建设。坚持不懈大搞农田水利基本建设，实施灌区续建配套与节水改造、预旺至下马关、大水坑节水补灌等水利骨干工程，新增高效节水灌溉16万亩，改造中低产田5万亩，兴修“三田”7万亩。认真实施天然林保护、退耕还林、三北防护林四期等生态建设工程，力争全年造林50万亩。五是继续深化农村改革。加快建立现代农村金融制度，扩大农业保险、村级互助资金试点范围，积极发展村镇银行。开展建立保障和促进科学发展土地管理新机制试点，探索实践农村土地流转、联保信贷改革，采取村企合作、社会运作等办法，引导农村土地承包经营权向农业龙头企业、农民专业合作社和种田能手集中，积极发展土地适度规模经营。

三、着力加快城乡一体化建设

按照自治区建设沿黄城市带，打造“黄河金岸”的要求，以规划管理为龙头，以项目建设为抓手，加快城乡一体化进程。力争新建续建城市建设项目314个，总投资78.7亿元。一是加强城乡规划管理。进一步完善城乡发展规划，编制市区西片区、青铜峡市小坝东区、同心县城新区、盐池县城新区及红寺堡、太阳山控制性详细规划，编制完善农村布局规划和庄点建设规划，实现城乡规划的全覆盖。加强规划审批，实行重大项目上管一级制度，严肃查处各类违法违规行为，坚决维护城乡规划的严肃性和权威性。把“两大工程”继续引向深入，完善长效机制，巩固提高城乡环境整治成果，全面提升园林绿化的档次和水平。加强市政设施、道路交通、市容市貌和社区物业管理，为争创“国家园林城市”、“全国卫生城市”奠定基础。二是推进重点项目建设。市区和青铜峡市加快推进黄河两岸30平方公里新区规划建设，实施吴忠黄河公路大桥、市区防洪排涝工程改造、城市道路、民俗博物馆、新月广场、教育园区、医疗垃圾废弃物集中处理、第二污水处理厂等项目。开工建设市区西片区城市规划道路，加快构筑城市快速交通网络，实现道路扩城；加快青铜峡小坝东区道路交通、文教卫生等重点公共基础设施建设，进一步拉大框架，完善功能；加快盐池县城东改西移、北控南扩，完成城南、城北生态水系建设，着力打造靓丽的宁夏东大门；推进同心县城新区3平方公里开发、骨干道路、供排水、供电供暖等基础设施建设，实施好清水河、北沟综合治理工程，着力打造干旱带水景观城；红寺堡、太阳山重点实施滚红高速公路、河东机场至太阳山高速公路太阳山过境段、省道101线南北段改造等道路工程，加快生态建设步伐，大面积植树铺绿，着力打造现代生态移民城市和水保生态工业城；加快金银滩、峡口、惠安堡、韦州等中心集镇的基础设施建设，逐步形成功能完善、环境优美、特色鲜明的城镇体系，积极引导各类资源向城镇合理流动。三是激活城市经营。加强土地管理，进一步整顿规范土地市场，完善土地登记制度，严格经营性土地招拍挂制度，垄断土地一级市场，依法管好盘活土地二级市场。全面完成第二次土地调查任务，加强基本农田保护和土地整理工作，确保占补平衡。深化投融资体制改革，积极探索建立以城市投资公司开发建设经营城市的新思路，加快国资经营集团公司运作步伐，盘活闲置国有资产和城市存量土地，采取土地入股、资本入股等方式，积极参与城市建设经营。加快城市派生资源经营步伐，采用托管经营、授权经营、股权转让等形式，逐步把垃圾处理、中水回用等可用市场经营的项目推向市场；加大城市无形资产开发利用，有偿转让城市户外广告标牌、公交线路经营权和城市设施冠名权等特许权，有效盘活城市存量资产。四是改善农村人居环境。认真实施农村道路、塞上农民新居、危窑危房改造工程，提高农村沼气、太阳能等清洁能源普及率。新建改建农村公路300公里，整治改造旧庄点72个，改造危窑危房1万户以上，建设“一池三改”户用沼气池9000座，太阳灶1.3万台。实施利通区孙家滩、青铜峡邵西、同心东部引水扩建、红寺堡中部饮水等农村饮水安全工程，解决5万人饮水安全问题。抓好第三批74个贫困村整村推进工作，大力实施同心闫圈、罗山东坡等生态移民工程，安置移民5万人。

四、着力提升第三产业发展水平

突出地方特色和优势，加快建设特色鲜明的现代商贸流通体系，培育壮大现代服务业，带动第三产业发展。一是加快发展现代商贸流通业。加快优势特色农产品市场建设，实施好“双百市场”工程，抓好东郊蔬菜批发市场和涝河桥牛羊肉批发市场改扩建、吴忠市综合建材城扩建、青铜峡仔猪市场、同心果蔬批发市场、盐池综合物流园区等农产品批发市场建设，完善商贸基础设施，力促国际商贸大厦上半年开业运营。完善城乡商业网点规划，大力发展连锁经营、物流配送、电子商务等新型业态，构建覆盖城乡的商贸流通网络。大力实施“三新工程”和“家电下乡”，抓好260个农家店的管理配送工作，提升“农家店”服务三农水平。不断完善农村流通体系，引导和鼓励有实力的商贸企业到农村发展，拓宽市场营销渠道，扩大农村消费，繁荣农村消费市场。二是壮大食品产业。加强餐饮单位规范管理和卫生监管，提升服务环境和服务质量。大力培育知名品牌和优势企业，鼓励餐饮名店走出去，发展连锁经营，培育一批在全区、全国叫得响的饮食店和名小吃连锁店，提升餐饮业档次。加快培育壮大食品加工产业，鼓励企业狠抓技术改造、设备更新、产品质量等环节，扩大生产、流通和销售，力争产值达到86亿元，增长15%。三是发展特色旅游业。大力推进黄河青铜峡大峡谷旅游区建设，按照打造宁夏又一王牌旅游景区的目标，加快108塔和库区湿地鸟岛开发，进一步提升景区接待能力；结合“生态旅游年”活动，加快整合提升市区、青铜峡黄河水上项目旅游圈，配套完善“农家乐”及民俗生态旅游景点基础设施，提升服务档次。完善盐池革命烈士纪念园、同心红军西征纪念馆和红寺堡生态移民博物院等景点功能，丰富红色旅游内容。深入推进“中国优秀旅游城市”创建活动。力争全年接待游客140万人次以上，旅游总收入突破5亿元，增长18%。四是引导房地产业健康发展。落实国家、自治区及我市房地产调控政策措施，继续加大房地产业扶持力度，以保障群众基本住房需求为导向，加强住房建设规划，调整住房供应结构，优先保证中低价位、中小户型普通商品房。积极引导和鼓励消费，在满足市场供应的同时，切实规范房地产市场秩序，加强市场交易和信息发布管理，搞活二手房市场，启动实施城市商网资源盘活工程，激活城市存量商业资源，力争通过适度的房地产开发建设、营业房盘活和二手房交易拉动消费增长。

五、着力推进和谐社会建设

加快科技创新。继续深化和高等院校、科研院所的技术交流与合作，加快工业新技术及农业新品种的开发应用。深入开展科技特派员创业行动和科技入户工程，积极推广节水灌溉、平衡施肥、绿色有机栽培等新技术，年内全市新建科技示范基地8个，培育农产品技术创新中心5个，科技特派员人数达到1000人，提高科技对经济增长的贡献率。新建市科技馆等一批科普场馆。办人民满意的教育。健全完善义务教育免费政策和经费保障机制，巩固提高“两基”成果，提升农村义务教育质量，逐步缩小城乡教育差距，不断推动义务教育均衡发展。盐池县实现教育强县创建目标。加快普及高中阶段教育，大力发展职业教育，重视学前教育，关注特殊教育，加强师生心理健康教育。进一步加大学校布局调整力度，优化教育资源配置，加强教育基础设施建设，完成教育园区宁夏民族职业技术学院、吴忠回民中学迁建任务，改扩建市区东塔中学、青铜峡二中、盐池高级中学、同心新区寄宿制初级中学、红寺堡回中等一批中小学校，着力解决中小学“大班额”、“大通铺”问题。提高劳动者素质。营造良好的人才成长环境，加大高层次、高技能人才引进和培养力度，努力开创人尽其才、人才辈出的发展局面。大力开展名校、名师、名校长评选活动，激发教育师资活力。加强劳动力转移就业技能培训，推动劳务输出由体力型向技能型转变，全年培训农民工4万人，输出劳务22万人次以上，实现劳务收入10亿元以上。发展文化体育事业。加强农村文化建设，继续扶持优秀文化艺术团体，广泛开展文化下乡、六进社区等群众喜闻乐见的文化宣传活动，更高水平办好第三届中国（吴忠）中小学生书法节。开展新中国成立60周年、建党88周年系列文化活动和红寺堡开发区建区十周年庆祝活动。加强公共文化服务基础设施建设，新建乡镇文体综合站26个、文化信息资源共享工程基层服务站6个、农家书屋50家，确保吴忠图书馆投入使用。加强文化市场监管，促进文化市场规范有序健康发展。积极做好文物和非物质文化遗产保护工作，深入挖掘、继承、发展特色文化，年内完成第二批自治区级非物质文化遗产代表作名录的申报工作。深入开展公民思想道德建设和群众性精神文明创建活动，加强社会主义核心价值体系建设，不断提高公民道德水平，形成良好的社会道德风尚。积极开展全民健身运动，办好吴忠市第二届运动会。

六、着力保障和改善民生

制定完善小额担保贷款与创业联动机制等相关政策，深入推进全民创业行动，按照“三个二六”目标，全年新培养小老板260个，新开发创业项目260个，新创造就业岗位2600个，以创业带动就业。加大就业培训、公益性岗位安置力度，力争全市新增城镇就业1.2万人，再就业1000人，援助零就业家庭就业300人，安置“4050”人员400人，城镇登记失业率控制在4.3%以内。完善失业、工伤、生育保险制度，切实做好失业保险扩面、征缴工作，力争养老、医疗、工伤、生育、失业保险参保人数达到26万人以上。实施村干部养老保险，建立失地农民养老保险制度，进一步完善和落实城乡居民最低生活保障等制度，重点做好失地农民就业与生活保障工作，力争应保尽保。加大城市廉租房、经济适用房建设力度，新建廉租房600套、经济适用住房3万平方米，力争将城市低收入家庭全部列入廉租房保障。继续实施市区“安居工程”建设，确保每一个城市拆迁户都能得到安置。进一步深化医疗卫生体制改革，完善药品“三统一”制度，健全覆盖城乡范围的公共卫生服务、医疗服务、医疗保障服务体系。全面推进城镇居民医疗保险，力争参保率达到90%以上。巩固和发展新型农村合作医疗工作，参合率达到90%以上。加强公共卫生基础设施建设，完成市中医院和11所乡镇中心卫生院等工程建设；积极争取市第二人民医院等项目尽快立项建设。加大社区卫生建设力度，深入推进农民健康教育与健康促进行动，促进全民保健。继续加强人口与计划生育工作，计划生育率达到85%以上。强化劳动保障监察，加大农民工工资清欠力度，维护农民工的合法权益。积极发展老年人服务和慈善事业，开工建设吴忠市残疾人综合服务中心、儿童福利院和流浪未成年人救助保护中心。加强残疾预防、残疾人康复工作，促进残疾人事业发展。加强社区建设，完成第七届社区居委会换届工作，构建和谐文明社区。

七、着力做好安全和维稳工作

加强公共安全。全面落实社会治安综合治理目标责任制，深化平安创建活动，建立健全防范网络，依法严厉打击各种刑事犯罪，进一步提高人民群众的安全感。坚持不懈地抓好禁毒工作，打好人民禁毒战争，巩固禁毒成果。重视加强消防安全、防震减灾工作，规划建设地震应急避难场所，切实做好建设工程消防监测监管、抗震设防监督管理。加强疫情预防预报、突发事件应对预案等体系建设，切实做好食品药品安全监管工作。加强安全生产。严格落实安全生产责任制和责任追究制，坚持不懈地抓好道路交通、矿山、危险化学品、建筑、公众聚集场所安全生产专项整治工作，坚决防止重特大安全生产事故的发生，确保人民群众生命财产安全。维护社会稳定。认真做好人民调解和信访工作，切实解决群众关心的热点、难点问题，努力把各类矛盾纠纷化解在萌芽状态。加强民兵预备役部队及人民防空建设，规划建设人防疏散基地和专业队伍训练基地，完善国防动员体系，增强全民国防意识，深入开展新一轮双拥模范城创建活动，提升工作水平。认真贯彻党的民族宗教政策，坚决抵御外来宗教的渗透，为全市经济社会发展创造和谐稳定的社会环境。

八、着力加强政府自身建设

把深入学习实践科学发展观活动与政府自身建设有机结合起来，牢固树立服务、责任、诚信和法治理念，加快转变政府职能，推进管理创新，提升服务水平。努力提高科学施政的能力。把与时俱进、开拓创新贯穿于政府工作全过程，开展调查研究，拓宽沟通民意渠道，问政于民、问需于民、问计于民，注重用基层的首创经验指导工作，提升科学发展的水平和层次。坚持把群众关心的热点、难点问题作为政府工作的重点，把群众需求作为政府工作的第一信号，把亲民、爱民、为民作为政府工作的基本准则，让群众从改革发展中得到更多的实惠。努力提高依法行政的能力。完善政府依法行政的决策规则和决策程序，健全权责明确、行为规范、监督有效、保障有力的行政执法体制，确保行政决策和执法行为符合法定程序，促进和约束行政执法人员严格、公正、文明执法，不断提高政府依法行政水平。进一步优化政务、司法、金融环境，大力整治投资环境，严厉打击欺生欺商、吃拿卡要等行为，保护客商正当利益和合法权益。严格按照法定的权限和程序履行职责，主动接受人大及其常委会的法律监督和人民政协的民主监督，自觉接受舆论监督、群众监督，认真办理人大代表议案、建议和政协委员提案，及时反馈办理情况。认真听取人大代表、政协委员、各民主党派、工商联、无党派人士和社会各界的意见建议，巩固扩大政务公开，提高政府工作的透明度和公信力。努力提高狠抓落实的能力。牢记党和人民的重托，肩负富民强市的重任，扎实工作，为民服务。稳步推进政府机构改革。启动市区农村综合改革试点工作，建立健全工作目标责任制，把经济社会发展的各项目标任务细化、量化，层层分解，进一步完善公务员考核激励机制、责任追究制和过失问责制，促进政府全体工作人员精心谋事、尽职办事，创造性地抓好落实，做到用新思路研究新问题，用新办法解决新矛盾，用新举措开创新局面。努力提高廉洁从政的能力。坚持标本兼治、综合治理、惩防并举、注重预防的方针，推进教育、制度、监督并重的惩治和预防腐败体系建设。深入开展机关效能建设，认真落实党风廉政建设责任制，落实领导干部“一岗双责”，切实抓好纠风和执法监察工作，坚决纠正损害群众利益的不正之风，切实从源头上预防和治理腐败，维护群众的切身利益。强化审计监督，严肃财经纪律，加强对重点领域、重点部门、重点资金的审计和行政机关、企事业单位主要负责人的经济责任审计，严肃查处违法违纪行为。牢记“两个务必”，坚决反对讲排场、比阔气、图享乐、奢侈浪费，树立为民、务实、清廉的政府形象。

各位代表！困难与希望同在，挑战和机遇并存。做好今年的各项工作，形势严峻，任务艰巨。让我们高举中国特色社会主义伟大旗帜，全面贯彻落实科学发展观，在自治区党委、政府和市委的坚强领导下，凝聚全市各族人民的智慧和力量，为全力推进我市经济社会又好又快发展而努力奋斗！
